# Severe mental illness as a risk factor for recorded diagnosis of osteoporosis and fragility fractures in people aged ≥50 years: retrospective cohort study using UK primary care data

**DOI:** 10.3399/BJGP.2024.0055

**Published:** 2024-10-15

**Authors:** Christina Avgerinou, Kate Walters, Juan Carlos Bazo-Alvarez, David Osborn, Robert Michael West, Andrew Clegg, Irene Petersen

**Affiliations:** Department of Primary Care and Population Health, University College London, London.; Department of Primary Care and Population Health, University College London, London.; Department of Primary Care and Population Health, University College London, London.; Division of Psychiatry, University College London, London; Camden and Islington NHS Foundation Trust, London.; Leeds Institute of Health Sciences, University of Leeds, Leeds.; Academic Unit for Ageing and Stroke Research, University of Leeds, Leeds.; Department of Primary Care and Population Health, University College London, London.

**Keywords:** electronic health records, fragility fracture, osteoporosis, primary health care, severe mental illness, bone density

## Abstract

**Background:**

Severe mental illness (SMI) has been associated with reduced bone density and increased risk of fractures, although some studies have shown inconsistent results.

**Aim:**

To examine the association between SMI and recorded diagnosis of osteoporosis and fragility fracture in people aged ≥50 years.

**Design and setting:**

Population-based cohort study set in UK primary care.

**Method:**

Anonymised primary care data (IQVIA Medical Research Database) were used. Patients with a diagnosis of SMI aged 50–99 years (2000–2018) were matched to individuals without SMI. Cox proportional hazards models were used to estimate hazard ratios (HRs) and 95% confidence intervals (CIs). Analyses were stratified by sex and age, accounting for social deprivation, year, smoking, alcohol, and body mass index.

**Results:**

In total, 444 480 people were included (SMI *n* = 50 006; unexposed *n* = 394 474). In men, diagnosis of SMI increased the likelihood of an osteoporosis diagnosis, with differences mainly observed among the youngest (aged 50–54 years: HR 2.12, 95% CI = 1.61 to 2.79) and the oldest (aged 85–99 years: HR 2.15, 95% CI = 1.05 to 4.37), and SMI increased the risk of fragility fractures across all ages. In women, SMI increased the risk of an osteoporosis diagnosis only in those aged 50–54 years (HR 1.16, 95% CI = 1.01 to 1.34), but increased the risk of fragility fractures across all ages. There were more than twice as many men with SMI with fragility fracture records than with an osteoporosis diagnosis: fragility fracture:osteoporosis = 2.10, compared with fragility fracture:osteoporosis = 1.89 in men without SMI. The fragility fracture:osteoporosis ratio was 1.56 in women with SMI versus 1.11 in women without SMI.

**Conclusion:**

SMI is associated with an increased likelihood of fragility fractures and osteoporosis underdiagnosis. Interventions should be considered to mitigate the increased risk of fractures in people with SMI.

## Introduction

The incidence of fractures has increased substantially in recent decades. Globally, in 2019, there were 178 million new fractures, 455 million prevalent cases of acute or long-term symptoms of a fracture, and 25.8 million years lived with disability.^1^ A significant proportion of these are fragility fractures, owing to osteoporosis. The global prevalence of osteoporosis is estimated at 18.3%, with a significantly higher prevalence in women.^2^ The economic burden of fragility fracture is significant, costing approximately £4 billion/year in the UK.^3^ Hip fractures are associated with the highest mortality and healthcare costs.^4^

Previous analysis of UK primary care data has demonstrated large differences in the incidence of fragility fracture by sex and age.^5^ The authors’ recent analysis of routinely collected primary care data demonstrated high incidence rates (IRs) of fragility fracture in the oldest age groups and women, underdiagnosis of osteopenia, and higher incidence of fragility fracture in people living in socially deprived areas, with remarkable effects in men.^6^

Severe mental illness (SMI) represents a spectrum of mental health diagnoses, including schizophrenia, bipolar disorder, and ‘other psychosis’, associated with significant mortality,^7^^–^^9^ disability,^10^^,^^11^ and health service costs.^12^ People with SMI are at a greater risk of poor physical health and have a higher premature mortality than the general population,^13^^,^^14^ and excess morbidity associated with social deprivation.^15^

Schizophrenia has been associated with reduced bone mineral density (BMD)^16^^,^^17^ and increased risk of fractures.^18^ It is not clear whether this association is as a result of antipsychotic medication, lifestyle factors, or both.^19^ Many antipsychotic drugs increase prolactin levels as a side effect, leading to an increase in osteoclast activity not compensated by osteoblast activity.^20^ In a 2012 systematic review, 15 of 16 studies reported lower BMD or higher prevalence of osteoporosis in at least one subgroup of patients with schizophrenia compared with those without schizophrenia, but results were inconsistent across measured areas; higher fracture risk was associated with schizophrenia in two of two studies and associated with antipsychotics in three of four studies.^21^ A 2007 UK case–control study using data from General Practice Research Database found that prolactin-raising antipsychotics were independently associated with hip fracture, but schizophrenia was not.^22^ On the contrary, a 2008 Canadian population-based study found that, although antipsychotics did not significantly increase the risk of osteoporotic fractures, schizophrenia diagnosis did in a fully adjusted model.^23^

Thus, there remains some discordance across studies regarding the association between fragility fractures and SMI. Most studies have focused on the use of antipsychotic medication and there is little research on the role of other factors. It is already known that SMI is associated with physical inactivity,^24^ poor nutrition,^25^ smoking,^26^ alcohol,^27^ and low vitamin D,^28^ which can all contribute to lower BMD. Moreover, there is little research on the recording of an osteoporosis diagnosis in people with SMI. Given additional barriers that people with SMI commonly face preventing them from seeking help,^29^ in the current study the authors hypothesised that BMD measurement and fracture risk assessment in primary care take place less often in people with SMI compared with the general population.

The objectives of the present study were: 1) to estimate the incidence of recorded osteoporosis diagnosis and fragility fractures in people with SMI aged ≥50 years in the UK; and 2) to compare the incidence of recorded osteoporosis diagnosis and fragility fractures between people aged ≥50 years with SMI and those without, accounting for age, sex, social deprivation, smoking, alcohol, and body mass index (BMI).

## Method

### Data source

Data provided as a part of routine primary care (IQVIA Medical Research Database [IMRD]) were used in the current study. Approximately 98% of the UK population is registered with a GP.^30^ The IMRD is a primary care database of >20 million patients in the UK, where GPs record medical diagnoses and symptoms using Read codes.^31^ All data are fully anonymised and representative of the UK population in terms of age, sex, practice size, and geographical distribution.^32^ Social deprivation is recorded in IMRD using the Townsend index, stratifying the population in quintiles of deprivation.^33^[Table table7]

**Table table7:** How this fits in

The physical health of people with severe mental illness (SMI) is often neglected, and this group of patients have higher rates of premature mortality. SMI has been associated with reduced bone mineral density and increased risk of fractures. This study has demonstrated that a diagnosis of SMI is a risk factor for fragility fractures in both men and women, accounting for age, social deprivation, smoking, alcohol, and body mass index. The study data suggest that osteoporosis is underdiagnosed both in men and women with SMI (with a relatively more pronounced effect in women with SMI compared with those without SMI), as well as in men without SMI. Interventions should be considered to screen for osteoporosis and mitigate the increased risk of fractures in people with SMI.

### Design

This was a longitudinal population-based cohort study.

### Study population

Patients aged ≥50 years registered with IMRD-participating practices between 1 January 2000 and 31 December 2018 who had a minimum of 12 months of follow-up data were included in the study. Practices that did not meet standards for data recording during the study period, that is, acceptable mortality reporting^34^ and acceptable computer usage, were excluded.^35^ Study entry was defined as the latest date of patient’s registration with the practice, when they turned 50 years old, or 1 January 2000. The start of the follow-up period was 12 months after study entry, thus individuals who died or left before the start of the follow-up period were excluded. The end of follow-up was set as the earliest of the outcome event date, the patient’s date of death, the patient’s transfer out of the practice, or the last date the practice contributed data to IMRD.

### Definition of variables

The explanatory variable was SMI, defined by a Read code of schizophrenia, bipolar disorder, or other psychosis (based on the authors’ previous validation study)^36^ (see Supplementary Information S1). The outcome variables were: 1) first recorded diagnosis of osteoporosis; and 2) first recorded fragility fracture, based on Read code (code lists published previously^6^). Prevalent cases of SMI were included (SMI diagnosed before outcome event). The demographic (age, Townsend quintile of deprivation, and calendar year) and lifestyle (smoking, alcohol, and BMI) covariates were treated as categorical variables (age: 5-year bands, year: 6-year intervals; the definition of lifestyle covariates are in Supplementary Table S1 and alcohol code list in Supplementary Information S2).

### Statistical analysis

Exposure density sampling (EDS) was used to identify a comparison cohort. This is an approach to dynamic matching with respect to a rare exposure occurring over time. For every exposed individual, unexposed individuals are sampled at the time of exposure from those who are still at risk of an event and not exposed at that point in time. Hence, a sample of individuals (yet unexposed) may be exposed after being sampled.^37^ This means that certain individuals served both as unexposed (before they were diagnosed with SMI) and exposed (from their SMI diagnosis onwards) at different time intervals. EDS was used to identify age- and sex-matched individuals (people with no prior SMI) within each GP practice at a 1:8 ratio. Two cohorts were produced, one for each outcome (recorded osteoporosis/fragility fracture). The reason behind this is that it is essential within the design of EDS to consider the event (or outcome) during the sampling process, because, *‘For every exposed individual, one samples controls at the time of exposure from those individuals who are still at risk for an event and still not exposed at that point in time.’*^37^

Crude IRs of recorded osteoporosis and fragility fracture in people with and without SMI per 10 000 person–years (PY) at risk was estimated by adding the number of patients with a first recording of osteoporosis or fragility fracture, and dividing by the total PY of follow-up. Moreover, the ratio between fragility fracture and osteoporosis diagnosis in people with/without SMI was calculated.

A fixed-effects Poisson model was compared against a mixed-effects Poisson model using GP practice as a random intercept. The Akaike’s and Bayesian information criterion were very similar with and without the GP cluster effect, therefore the GP practice was not included in the model.

Interactions between age/sex, age/exposure, and sex/exposure were tested. Analyses were stratified by sex and age, and Cox proportional hazard regression models were used to estimate hazard ratios (HRs) and 95% confidence intervals (CIs). The proportional hazard assumption was met using the proportional hazard test. Age-specific HRs were estimated. Unadjusted and adjusted analyses were conducted: model 1 adjusted for Townsend quintile of deprivation and year and model 2 adjusted for model 1 covariates plus smoking, alcohol, and BMI.

Supplementary subgroup analyses were undertaken to compare risk of osteoporosis/fragility fracture by SMI type (schizophrenia, bipolar, and other psychosis) adjusted by age, deprivation, and year.

Statistical analyses were conducted using Stata (version 17).

### Missing data

There were no missing data for age, sex, and Townsend. However, only 35% of the cohort had full data on all three of smoking, alcohol, and BMI, whereas for the remaining 65% ≥1 of these were missing. People with SMI had fewer missing data compared with those without SMI on smoking (14.7% versus 26.9%), alcohol (40.9% versus 61.4%), and BMI (29.2% versus 42.2%) (see Supplementary Table S2), but there were no significant differences in missing data between men and women (see Supplementary Table S3). Further information about missingness on smoking, alcohol, and BMI by sex and age is presented in Supplementary Tables S4, S5, and S6, respectively. Multiple imputation^35^ was performed for smoking, alcohol, and BMI to obtain estimates under the missing at random assumption (multiple imputation details in Supplementary Information S3).

## Results

### Study cohort

The SMI osteoporosis cohort consisted of a total 444 480 people (50 006 exposed, 397 474 age- and sex-matched unexposed individuals, among whom 1437 served in both groups at different time intervals, as explained above) (see Supplementary Table S7). The SMI fragility fracture cohort consisted of a total 425 364 people (47 851 exposed and 377 513 unexposed, among whom 1351 served in both groups at different time intervals) (see Supplementary Table S8). The ratio of exposed to unexposed was 1 to 7.9 (with a very small discrepancy to the intended 1:8 because of the complexity of EDS that was used for the matching of individuals).

Significant interactions were found between age and sex both for osteoporosis (*P* = 0.0190) and for fragility fracture (*P*<0.0001), between age and exposure for osteoporosis (*P* = 0.0001), but not for fragility fracture (*P* = 0.3365), and between sex and exposure for osteoporosis (*P*<0.0001) and fragility fracture (*P*<0.0001) (see Supplementary Figures S1–S6). Stratified analyses were performed in view of these interactions.

### IR of recorded osteoporosis and fragility fracture in people with versus without SMI

The IRs of an osteoporosis diagnosis were estimated at 22.99 (95% CI = 20.28 to 25.97)/10 000 PY in men and 76.36 (95% CI = 72.04 to 80.88)/10 000 PY in women with SMI versus 15.21 (95% CI = 14.50 to 15.95)/10 000 PY in men and 79.48 (95% CI = 78.07 to 80.92)/10 000 PY in women without SMI. The IRs of fragility fractures were 48.33 (95% CI = 44.27 to 52.67)/10 000 PY in men and 119.46 (95% CI = 113.94 to 125.18)/10 000 PY in women with SMI versus 28.70 (95% CI = 27.69 to 29.73)/10 000 PY in men and 88.12 (95% CI = 86.60 to 89.65)/10 000 PY in women without SMI (Tables 1 and 2).

**Table 1.** IRs of recorded osteoporosis diagnosis and fragility fractures in people aged ≥50 years with a diagnosis of SMI stratified by sex (2001–2018)

**Characteristic**	**Men**			**Women**		

	**Recorded** **osteoporosis** **diagnosis, IR per** **10 000 PY (95%** **CI)**	**Recorded fragility** **fracture, IR per** **10 000 PY (95% CI)**	**Ratio** **fragility** **fracture:** **osteoporosis** **diagnosis**	**Recorded** **osteoporosis** **diagnosis, IR per** **10 000 PY (95% CI)**	**Recorded fragility** **fracture, IR per** **10 000 PY (95% CI)**	**Ratio** **fragility** **fracture:** **osteoporosis** **diagnosis**
**Age, years**						
All ages	22.99 (20.28 to 25.97)	48.33 (44.27 to 52.67)	2.10	76.36 (72.04 to 80.88)	119.46 (113.94 to 125.18)	1.56
50–54	13.76 (9.28 to 19.65)	19.18 (13.70 to 26.11)	1.39	29.05 (22.47 to 36.96)	40.81 (32.86 to 50.10)	1.40
55–59	15.23 (10.73 to 21.00)	30.56 (23.87 to 38.55)	2.01	51.11 (42.91 to 60.42)	53.67 (45.18 to 63.30)	1.05
60–64	19.25 (13.75 to 26.21)	34.22 (26.57 to 43.38)	1.78	58.48 (49.20 to 69.01)	68.04 (57.90 to 79.44)	1.16
65–69	28.84 (21.27 to 38.24)	43.87 (34.20 to 55.43)	1.52	78.95 (67.43 to 91.87)	106.54 (92.93 to 121.59)	1.35
70–74	26.89 (18.39 to 37.96)	64.85 (50.92 to 81.41)	2.41	109.45 (94.67 to 125.90)	137.16 (120.36 to 155.66)	1.25
75–79	42.18 (29.38 to 58.67)	89.38 (69.81 to 112.74)	2.12	137.37 (119.25 to 157.47)	198.04 (175.71 to 222.41)	1.44
80–84	37.43 (22.87 to 57.81)	141.02 (110.34 to 177.59)	3.77	110.46 (92.42 to 130.99)	236.49 (208.87 to 266.75)	2.14
85–99	47.47 (27.65 to 76.00)	161.05 (121.33 to 209.64)	3.39	92.58 (76.76 to 110.70)	315.58 (283.60 to 350.18)	3.41

**Townsend quintile**						
1 (least deprived)	27.52 (20.36 to 36.38)	55.88 (45.27 to 68.24)	2.03	81.93 (71.72 to 93.18)	126.36 (113.41 to 140.38)	1.54
2	19.77 (14.19 to 26.82)	42.07 (33.56 to 52.09)	2.13	77.33 (67.80 to 87.83)	123.43 (111.07 to 136.78)	1.60
3	23.95 (18.04 to 31.18)	47.23 (38.55 to 57.29)	1.97	75.24 (66.08 to 85.32)	119.52 (107.68 to 132.30)	1.59
4	17.94 (13.13 to 23.93)	48.86 (40.47 to 58.46)	2.72	67.79 (59.26 to 77.20)	119.72 (108.09 to 132.26)	1.77
5 (most deprived)	26.68 (20.72 to 33.83)	48.60 (40.26 to 58.15)	1.82	81.29 (71.01 to 92.63)	107.59 (95.50 to 120.78)	1.32

**Year**						
2001–2006	19.69 (14.83 to 25.63)	41.58 (34.23 to 50.03)	2.11	75.71 (67.70 to 84.41)	111.68 (101.70 to 122.38)	1.48
2007–2012	22.04 (17.93 to 26.81)	46.18 (40.01 to 53.02)	2.10	80.37 (73.45 to 87.76)	125.52 (116.69 to 134.83)	1.56
2013–2018	26.45 (21.61 to 32.04)	55.72 (48.41 to 63.83)	2.11	71.78 (64.38 to 79.79)	118.55 (108.83 to 128.90)	1.65

*IR = incidence rate. PY = person-years. SMI = severe mental illness.*

**Table 2.** IRs of recorded osteoporosis diagnosis and fragility fractures in people aged ≥50 years with no diagnosis of SMI (unexposed) stratified by sex (2001 to 2018)

**Characteristic**	**Men**			**Women**		

	**Recorded osteoporosis diagnosis, IR per 10 000 PY (95% CI)**	**Recorded fragility fracture, IR per 10 000 PY (95% CI)**	**Ratio fragility fracture: osteoporosis diagnosis**	**Recorded osteoporosis diagnosis, IR per 10 000 PY (95% CI)**	**Recorded fragility fracture, IR per 10 000 PY (95% CI)**	**Ratio fragility fracture: osteoporosis diagnosis**
**Age, years**						
All ages	15.21 (14.50 to 15.95)	28.70 (27.69 to 29.73)	1.89	79.48 (78.07 to 80.92)	88.12 (86.60 to 89.65)	1.11
50–54	3.82 (2.98 to 4.83)	12.80 (11.18 to 14.60)	3.35	23.99 (21.82 to 26.32)	24.35 (22.14 to 26.71)	1.02
55–59	6.99 (5.93 to 8.19)	15.42 (13.79 to 17.20)	2.21	38.39 (35.92 to 40.99)	37.09 (34.65 to 39.66)	0.97
60–64	9.99 (8.65 to 11.48)	17.14 (15.32 to 19.10)	1.72	58.48 (55.31 to 61.80)	46.51 (43.67 to 49.49)	0.80
65–69	16.93 (15.00 to 19.04)	22.56 (20.28 to 25.03)	1.33	79.98 (76.08 to 84.03)	66.22 (62.66 to 69.92)	0.83
70–74	19.06 (16.73 to 21.62)	29.46 (26.48 to 32.68)	1.55	105.36 (100.60 to 110.29)	86.31 (82.00 to 90.80)	0.82
75–79	26.63 (23.51 to 30.04)	44.24 (40.11 to 48.69)	1.66	126.28 (120.82 to 131.93)	128.92 (123.36 to 134.67)	1.02
80–84	37.59 (33.22 to 42.37)	67.50 (61.46 to 73.99)	1.80	136.36 (130.37 to 142.55)	169.31 (162.46 to 176.37)	1.24
85–99	39.13 (34.28 to 44.48)	109.61 (101.12 to 118.63)	2.80	101.70 (97.29 to 106.27)	202.65 (195.96 to 209.51)	1.99

**Townsend quintile**						
1 (least deprived)	13.62 (12.28 to 15.06)	25.12 (23.25 to 27.09)	1.84	75.88 (73.13 to 78.71)	81.16 (78.28 to 84.12)	1.07
2	13.10 (11.75 to 14.56)	26.58 (24.60 to 28.68)	2.03	73.37 (70.57 to 76.25)	85.93 (82.87 to 89.08)	1.17
3	16.64 (15.03 to 18.38)	29.15 (26.96 to 31.47)	1.75	80.30 (77.24 to 83.44)	88.38 (85.10 to 91.75)	1.10
4	15.71 (14.03 to 17.55)	30.98 (28.53 to 33.59)	1.97	82.18 (78.81 to 85.67)	92.57 (88.94 to 96.31)	1.13
5 (most deprived)	18.99 (16.83 to 21.35)	35.45 (32.41 to 38.69)	1.87	93.59 (89.17 to 98.18)	99.99 (95.34 to 104.81)	1.07

**Year**						
2001–2006	12.64 (11.28 to 14.12)	24.17 (22.23 to 26.24)	1.91	84.29 (81.39 to 87.27)	72.99 (70.24 to 75.83)	0.87
2007–2012	13.96 (12.91 to 15.08)	27.04 (25.53 to 28.62)	1.94	79.97 (77.77 to 82.22)	94.74 (92.30 to 97.22)	1.18
2013–2018	18.12 (16.85 to 19.46)	33.27 (31.49 to 35.12)	1.84	75.28 (72.91 to 77.71)	91.10 (88.45 to 93.81)	1.21

*IR = incidence rate. PY = person-years. SMI = severe mental illness.*

In general, it was observed that there were more than twice as many men with SMI with a fragility fracture record than those diagnosed with osteoporosis (fragility fracture:osteoporosis = 2.10). This ratio was slightly smaller in men without SMI (fragility fracture:osteoporosis = 1.89). In women with SMI the fragility fracture:osteoporosis ratio was 1.56, whereas in women without SMI the ratio was 1.11 (Tables 1 and 2).

### Recorded osteoporosis diagnosis

In men, diagnosis of SMI increased the likelihood of a recorded osteoporosis diagnosis in specific age groups but not in others. In the fully adjusted model, differences were primarily observed among the younger (aged 50–54 years: HR 2.12, 95% CI = 1.61 to 2.79) and older age groups (aged 85–99 years: HR 2.15, 95% CI = 1.05 to 4.37) (Table 3).

**Table 3.** Association between diagnosis of SMI and recorded osteoporosis diagnosis in men: age-specific HRs for men with SMI versus men without SMI

**Age, years**	***N***	**Unadjusted**		**Adjusted model** 1**^a^**		**Adjusted model** 2**^b^**	

		**HR**	**95% CI**	**HR**	**95% CI**	**HR**	**95% CI**
50–54	78 942	2.52	1.95 to 3.25	2.34	1.81 to 3.03	2.12	1.61 to 2.79
55–59	29 596	1.45	1.04 to 2.03	1.39	1.00 to 1.94	1.16	0.82 to 1.65
60–64	22 874	1.77	1.27 to 2.48	1.67	1.19 to 2.35	1.44	1.01 to 2.05
65–69	18 485	1.67	1.17 to 2.40	1.59	1.10 to 2.28	1.50	1.03 to 2.18
70–74	14 507	1.40	0.94 to 2.11	1.39	0.92 to 2.08	1.25	0.82 to 1.90
75–79	12 694	1.41	0.94 to 2.11	1.38	0.92 to 2.07	1.29	0.85 to 1.96
80–84	8529	0.81	0.40 to 1.66	0.81	0.40 to 1.66	0.80	0.39 to 1.65
85–99	5249	2.22	1.13 to 4.37	2.27	1.15 to 4.47	2.15	1.05 to 4.37

a *Adjusted for Townsend and calendar year (6-year intervals).*

b *Adjusted for Townsend, calendar year (6-year intervals), smoking, alcohol, and body mass index. Missing data on smoking, alcohol, and body mass index were handled with multiple imputation for bias correction. HR = hazard ratio. SMI = severe mental illness.*

In women, age-specific HRs showed only a slightly increased risk of osteoporosis diagnosis associated with SMI in those aged 50–54 years (HR 1.16, 95% CI = 1.01 to 1.34) and less likely in the age group 80–84 years (HR 0.74, 95% CI = 0.59 to 0.93), with no relative differences in other age groups (Table 4).

**Table 4.** Association between diagnosis of SMI and recorded osteoporosis diagnosis in women: age-specific HRs for women with SMI versus women without SMI

**Age, years**	***N***	**Unadjusted**		**Adjusted model** 1**^a^**		**Adjusted model** 2**^b^**	

		**HR**	**95% CI**	**HR**	**95% CI**	**HR**	**95% CI**
50–54	80 139	1.19	1.03 to 1.37	1.17	1.02 to 1.35	1.16	1.01 to 1.34
55–59	31 052	1.16	0.98 to 1.36	1.13	0.96 to 1.34	1.11	0.93 to 1.31
60–64	26 391	0.96	0.81 to 1.13	0.95	0.80 to 1.12	0.95	0.80 to 1.12
65–69	24 576	1.01	0.87 to 1.18	1.00	0.86 to 1.16	0.97	0.83 to 1.14
70–74	22 968	1.04	0.89 to 1.21	1.02	0.88 to 1.20	1.01	0.86 to 1.18
75–79	22 823	0.88	0.74 to 1.06	0.88	0.73 to 1.05	0.87	0.72 to 1.04
80–84	21 481	0.75	0.59 to 0.94	0.75	0.59 to 0.94	0.74	0.59 to 0.93
85–99	24 174	1.05	0.82 to 1.35	1.04	0.81 to 1.34	1.00	0.78 to 1.29

a *Adjusted for Townsend and calendar year (6-year intervals).*

b *Adjusted for Townsend, calendar year (6-year intervals), smoking, alcohol, and body mass index. Missing data on smoking, alcohol, and body mass index were handled with multiple imputation for bias correction. HR = hazard ratio. SMI = severe mental illness.*

### Recorded fragility fracture

In men, diagnosis of SMI increased the risk of fragility fractures across all age groups, albeit with some small variation. In the fully adjusted model, this risk ranged from HR 1.52 (95% CI = 1.23 to 1.88) in men aged 50–54 years up to HR 2.29 (95% CI = 1.78 to 2.96) in men aged 65–69 years and HR 2.14 (95% CI = 1.55 to 2.94) in men aged 80–84 years (Table 5).

**Table 5.** Association between diagnosis of SMI and recorded fragility fracture in men: age-specific HRs for men with SMI versus men without SMI

**Age, years**	***N***	**Unadjusted**		**Adjusted model** 1**^a^**		**Adjusted model** 2**^b^**	

		**HR**	**95% CI**	**HR**	**95% CI**	**HR**	**95% CI**
50–54	75 610	1.71	1.40 to 2.09	1.59	1.30 to 1.94	1.52	1.23 to 1.88
55–59	28 317	1.78	1.38 to 2.30	1.64	1.27 to 2.11	1.56	1.19 to 2.05
60–64	22 108	2.14	1.65 to 2.78	2.03	1.56 to 2.64	1.95	1.48 to 2.58
65–69	17 627	2.44	1.91 to 3.11	2.36	1.85 to 3.01	2.29	1.78 to 2.96
70–74	13 897	1.91	1.46 to 2.50	1.90	1.45 to 2.49	1.92	1.45 to 2.55
75–79	12 164	1.77	1.34 to 2.34	1.77	1.33 to 2.34	1.72	1.29 to 2.30
80–84	8025	2.10	1.55 to 2.84	2.08	1.53 to 2.83	2.14	1.55 to 2.94
85–99	4815	1.97	1.26 to 3.06	1.96	1.26 to 3.06	1.81	1.15 to 2.85

a *Adjusted for Townsend and calendar year (6-year intervals).*

b *Adjusted for Townsend, calendar year (6-year intervals), smoking, alcohol, and body mass index. Missing data on smoking, alcohol, and body mass index were handled with multiple imputation for bias correction. HR = hazard ratio. SMI = severe mental illness.*

In women, diagnosis of SMI increased the risk of fragility fractures across all age groups, with some small variation of the risk ranging from HR 1.32 (95% CI = 1.15 to 1.52) in those aged 70–74 years up to HR 1.80 (95% CI = 1.56 to 2.08) in those aged 80–84 years in the fully adjusted model (Table 6).

**Table 6.** Association between diagnosis of SMI and recorded fragility fracture in women: age-specific HRs for women with SMI versus women without SMI

**Age, years**	***N***	**Unadjusted**		**Adjusted model** 1**^a^**		**Adjusted model** 2**^b^**	

		**HR**	**95% CI**	**HR**	**95% CI**	**HR**	**95% CI**
50–54	78 593	1.40	1.23 to 1.60	1.36	1.19 to 1.55	1.35	1.18 to 1.56
55–59	30 515	1.77	1.53 to 2.05	1.77	1.53 to 2.05	1.73	1.49 to 2.02
60–64	25 978	1.52	1.30 to 1.76	1.50	1.29 to 1.74	1.48	1.26 to 1.73
65–69	24 116	1.52	1.32 to 1.74	1.52	1.32 to 1.75	1.49	1.29 to 1.71
70–74	22 338	1.37	1.20 to 1.57	1.36	1.18 to 1.55	1.32	1.15 to 1.52
75–79	21 517	1.43	1.24 to 1.64	1.43	1.24 to 1.64	1.41	1.23 to 1.62
80–84	19 587	1.81	1.57 to 2.08	1.82	1.58 to 2.09	1.80	1.56 to 2.08
85–99	20 157	1.64	1.41 to 1.91	1.63	1.40 to 1.90	1.61	1.38 to 1.88

a *Adjusted for Townsend and calendar year (6-year intervals).*

b *Adjusted for Townsend, calendar year (6-year intervals), smoking, alcohol, and body mass index. Missing data on smoking, alcohol, and body mass index were handled with multiple imputation for bias correction. HR = hazard ratio. SMI = severe mental illness.*

Overall, the HRs show an increased risk of a fragility fracture record for people with SMI (both men and women). There are small variations in those HRs across age groups, but the results are quite consistent, with little evidence of interaction between age and exposure (as shown above) for the outcome of fragility fracture.

Complete case analysis results are presented in Supplementary Tables S9 and S10. Comparison of these against the imputed results (Tables 3–6) shows that the HRs were quite similar, but the CIs were narrower for the analyses based on imputed data.

### Effect of SMI subtype

Men in the 50–54 years age group with schizophrenia (HR 1.68, 95% CI = 1.03 to 2.75) and bipolar disorder (HR 2.15, 95% CI = 1.28 to 3.62) were more likely to receive an osteoporosis diagnosis compared with men without SMI. Men with other psychosis were more likely to be diagnosed with osteoporosis aged 50–54 years (HR 2.81, 95% CI = 2.03 to 3.89) compared with those without SMI, with small differences in some other age groups (60–64 years, 65–69 years, and 70–74 years) (see Supplementary Table S11).

Women with other psychosis in their 50s had a slightly higher chance of being diagnosed with osteoporosis compared with women without SMI (aged 50–54 years: HR 1.29, 95% CI = 1.08 to 1.55; aged 55–59 years: HR 1.28, 95% CI = 1.03 to 1.59), whereas there were no significant differences for other SMI subtypes or age groups (see Supplementary Table S12).

Men and women with all subtypes of SMI were at an increased risk of fragility fractures across most (but not all) age groups compared with those without SMI, with variation of the observed risk by age (see Supplementary Tables S13 and S14).

## Discussion

### Summary

The current findings suggest that SMI is an independent risk factor for fragility fractures across all age groups in both men and women, accounting for social deprivation, smoking, alcohol, and BMI. Men with SMI are more likely to be diagnosed with osteoporosis if they are in their early 50s or above 85 years of age. Women with SMI are more likely to be diagnosed with osteoporosis in their early 50s and less likely in their early 80s, with no relative differences in other age groups. Among men with SMI there were more than twice as many with a fragility fracture record than with an osteoporosis diagnosis. This ratio was slightly smaller for men without SMI (fragility fracture:osteoporosis = 1.89). For women with SMI the fragility fracture:osteoporosis ratio was 1.56, whereas for women without SMI the ratio was 1.11. These figures suggest that osteoporosis is underdiagnosed both in men and women with SMI (with a relatively more pronounced effect in women with SMI compared with those without SMI), as well as in men without SMI.

### Strengths and limitations

The main strength of this study is the robust methodology, using nationally representative, real-world data. A limitation is that analyses were based on Read codes as they were recorded by GPs, which can be influenced by various factors.^38^ The diagnosis of SMI is traditionally established by a psychiatrist, and the classification is expected to follow current guidelines at the time of diagnosis, some of which may have changed during the study period.^39^ There was no access to dual-energy X-ray absorptiometry results in the current study, therefore the incidence of osteoporosis is likely to be underestimated. The high proportion of missing data on smoking, alcohol, and BMI was addressed through multiple imputation, which is a reliable method to reduce bias.^40^ After adjusting for smoking, alcohol, and BMI, the current study found an increased risk of fractures in people with SMI. Given the negative impact of antipsychotic medication on BMD mentioned above, this might be because of antipsychotic medication. However, investigating the effect of medication was not undertaken at this time, as it was outside the scope of this project and would require a different study design. Finally, data were not available regarding other lifestyle factors, for example, physical activity, which can also affect BMD.

### Comparison with existing literature

The IR of recorded osteoporosis in men with SMI in the current study (22.99 [95% CI = 20.28 to 25.97]/10 000 PY) was higher compared with the IR previously reported in the general population of men aged ≥50 years (15.28 [95% CI = 15.06 to 15.51]/10 000 PY).^6^ In contrast, the IR of recorded osteoporosis in women with SMI (76.36 [95% CI = 72.04 to 80.88]/10 000 PY) was slightly lower but similar compared with that in the general population of women aged ≥50 years (79.82 [95% CI = 79.32 to 80.31]/10 000 PY).^6^ The IRs of fragility fractures in people with SMI in the current study were much higher compared with the general population aged ≥50 years (men: 48.33 [95% CI = 44.27 to 52.67]/10 000 PY versus 28.72 [95% CI = 28.41 to 29.03]/10 000 PY; women: 119.46 [95% CI = 113.94 to 125.18]/10 000 PY versus 82.01 [95% CI = 81.50 to 82.51]/10 000 PY).^6^ The IRs of recorded osteoporosis and fragility fracture in people without SMI were very similar to those reported in the general population aged ≥50 years.^6^

Another study using data from South London and Maudsley (SLaM) NHS Biomedical Research Centre Case Register (2006–2012) found that increasing age, White ethnicity, analgesics, cardiovascular disease, hypertension, genitourinary diseases, visual disturbance, and syncope were significant risk factors for both falls and fractures in people with schizophrenia-spectrum disorders.^41^ A Canadian study showed that antipsychotic medication increased the risk of hip fracture above and beyond risk factors included in the FRAX risk assessment, whereas FRAX underestimated the 10-year risk of hip fracture in people taking selective serotonin reuptake inhibitors, mood stabilisers, antipsychotics, or benzodiazepines.^42^ A population-based cohort study from Taiwan that included a younger population (aged ≥16 years) found that people with bipolar disorder had a higher risk of fracture HR 1.33 (95% CI = 1.23 to 1.48) compared with those without bipolar. The risk increased by age, although the results were adjusted for but not stratified by sex.^43^

Another study using linked primary (Lambeth DataNet) and secondary care data (SLaM), which included all adults (aged ≥18 years), reported that people with SMI were more likely to be prescribed medication for osteoporosis and be referred for osteoporosis screening within 2 years of SMI diagnosis, after adjusting for ethnicity, deprivation, and comorbidities, which was an unexpected finding. The authors hypothesised that the reason behind this might be the higher levels of comorbidity in people with SMI leading to more engagement with primary care.^44^ Moreover, other UK studies have found an increased risk of falls requiring admission to hospital among adults of working age receiving mental health care (aged 18–64 years),^45^ and a two-fold risk of falls and four-fold risk of hip fracture in people aged >60 years receiving mental health care.^46^ In the above-mentioned studies, analyses were stratified by age, but no age- or sex-specific risks were presented, therefore their results cannot be compared with the current study. Moreover, to the authors’ knowledge, previous studies have not investigated osteoporosis diagnosis in people with SMI based on primary care data.

In the current study some age differences were found regarding diagnosis of osteoporosis. More specifically, the HR for recorded osteoporosis diagnosis in men is greatest for those aged 50–54 years and those aged 85–99 years, but lower for the intervening age groups, comparing individuals with SMI versus those without. These differences are likely to represent gaps in osteoporosis screening in the youngest and oldest age groups in men without SMI. Physical health checks in middle age (50 years) might trigger the identification of risk factors for osteoporosis. Presence of SMI is more likely to be associated with multiple comorbidities in older age, and as a result hospital admissions may also trigger more investigations including for osteoporosis (for example, risk of falls). The current study interestingly found that women with SMI aged 80–84 years are less likely to be diagnosed with osteoporosis compared with those without SMI. Factors such as social isolation, frailty, and dementia (which are more common in women) might affect osteoporosis screening, although it is not possible to determine these associations with certainty within the current dataset. It may, however, be that recording of fragility fractures (as a hard outcome) is a more reliable indicator of bone health compared with recorded osteoporosis, which is often not diagnosed until a fracture occurs.

### Implications for research and practice

The above findings indicate an increased risk of fragility fractures in people aged ≥50 years with a diagnosis of SMI. It is not clear if this difference could be because of antipsychotic medication, an underlying biological mechanism of an association between osteoporosis and SMI, other factors such as lack of exercise, or differences in osteoporosis management. Further research is needed to explore inequalities in osteoporosis screening and treatment in the presence of SMI.

Primary care clinicians need to become aware of the increased fracture risk in people with SMI, which could be addressed during/following physical health checks. Fracture risk assessment and appropriate osteoporosis treatment as indicated may need to be included in the annual comprehensive care plan in people with SMI. Advice on diet and resistance training to prevent osteoporosis and fractures should also be evaluated.

In conclusion, SMI is associated with increased risk of fragility fractures in both men and women, and osteoporosis is underdiagnosed in people with SMI. Osteoporosis screening and management may need to be considered as part of the annual care plan for individuals with SMI. Appropriate interventions to prevent fragility fractures in people with SMI are needed.
